# The potential risk factors of early-onset post-stroke depression from immuno-inflammatory perspective

**DOI:** 10.3389/fimmu.2022.1000631

**Published:** 2022-09-26

**Authors:** Hengshu Chen, Fan Liu, Dongren Sun, Jingyuan Zhang, Shihang Luo, Qiao Liao, Fafa Tian

**Affiliations:** Department of Neurology, Xiangya Hospital, Central South University, Changsha, China

**Keywords:** post-stoke depression, single nucleotide polymorphisms, splenic attenuation, splenic volume, immuno-inflammatory response

## Abstract

**Background:**

Mounting evidence strongly uncovered that peripheral immuno-inflammatory response induced by acute stroke is associated with the appearance of post-stroke depression (PSD), but the mechanism remains unclear.

**Methods:**

103 stroke patients were assessed at 2 weeks after onset using Diagnostic and Statistical Manual of Mental Disorders, 5th edition and then divided into PSD and non-PSD groups. Polymorphisms of inflammatory molecules (interleukin [IL]-1β, IL-6, IL-10, IL-18, tumor necrosis factor-α [TNF-α], interferon-γ [IFN-γ] and C-reactive protein [CRP]), complete blood count parameters, splenic attenuation (SA) and splenic volume (SV) on unenhanced chest computed tomography, demographic and other clinical characteristics were obtained. Binary logistic regression model was used to analyze the associations between inflammation-related factors and the occurrence of PSD at 2 weeks after stroke.

**Results:**

49 patients were diagnosed with PSD at 2 weeks after onset (early-onset PSD). The C/T genotypes of CRP rs2794520 and rs1205 were less in PSD group than non-PSD group (both adjusted odds ratio = 3.364; 95%CI: 1.039-10.898; *p* = 0.043). For CRP rs3091244, the frequency of G allele was higher (80.61% vs. 13.89%) while the frequency of A allele was lower (6.12% vs. 71.30%) in PSD patients than non-PSD patients (χ^2^ = 104.380; *p*<0.001). SA of PSD patients was lower than that of non-PSD patients in the presence of CRP rs2794520 C/T genotype and rs1205 C/T genotype (both t = 2.122; *p* = 0.039). Peripheral monocyte count was less in PSD group than non-PSD group (adjusted odds ratio = 0.057; 95%CI: 0.005-0.686; *p* = 0.024).

**Conclusions:**

CRP polymorphisms, SA based on CRP genotype, and peripheral monocytes are associated with the risk of early-onset PSD, suggesting peripheral immuno-inflammatory activities elicited by stroke in its aetiology.

## Introduction

Post-stroke depression (PSD), one of the most prevalent complications of stroke, afflicts approximately 31% of stroke survivors (1). The emergence of depression after stroke is closely coupled with further worsening of functional ability and quality of life (2), low rehabilitation efficiency (3), increased disability and mortality (2). Due to the lack of reliable biomarkers, the early diagnosis of PSD is still difficult (4). The main reason may be that the mechanisms responsible for the etiology of PSD have, so far, not yet been undetermined. In recent years, many scholars have supported the important role of inflammation in the risk of PSD. The available data have shown that immuno-inflammatory responses are activated immediately after acute stroke, both centrally and peripherally, followed by impressive increased expression of pro-inflammatory cytokines (such as interleukin [IL]-1, IL-6 and tumor necrosis factor [TNF]-α) to initiate and/or amplify inflammatory response (5), causing dysfunction of noradrenergic system, hyperactivity of the hypothalamic pituitary adrenal (HPA) axis and extensive activation of indoleamine 2,3-dioxygenase enzyme that accelerates serotonin depletion in physiological regions such as paralimbic areas of left frontal and temporal cortex, which may ultimately lead to depression (6–9). Although there is a large amount of evidence that inflammatory molecules are involved in the formation of PSD (10–13), but genetic factors underlying the association remain unclear.

Single nucleotide polymorphism (SNP) is a common genetic variation and has become main molecular genetic marker conducive to our understanding of diseases with genetic susceptibility (14). It has been suggested that the production of inflammatory molecules is influenced by the transcriptional activity of gene polymorphisms (15). Exploring the associations between inflammatory molecule gene SNPs and PSD have significant implications for further understanding immuno-inflammatory mechanism of PSD. However, to our knowledge, there is considerably a paucity of research on the influence of inflammation-related genetic factors on propensity to PSD.

It is believed that activation of peripheral immunity and secondary intracerebral neuroinflammation after stroke are mainly the result of brain-spleen communication (16–18). The spleen is a major lymph organ containing an abundance of immunological cells, which can rapidly deploy the distribution of immune cells in the system to fight injury including brain insult (19). The spleen is activated soon after stroke onset and resident immune cells such as monocytes, lymphocytes, and neutrophils are released into the bloodstream, leading to increased secretion of cytokines and the spleen shrink, and subsequently these cells carrying copious inflammatory mediators migrate into the damaged brain, thereby exacerbating the local brain inflammation during the process of stroke (16, 20, 21). These findings suggest that stroke not only triggers changes in splenic components (that is, splenocyte redistribution and abnormal cytokines expression, which may influence splenic density), but also changes in splenic volume (SV). Spleen appears to be a crucial pivot linking stroke to PSD, considering its ability in the initiation and amplification of immuno-inflammatory response.

Taking into account the role that immune-inflammation system plays in the occurrence of PSD and given the sparse evidence on inflammation-related genetic factor of PSD. The current study, therefore, investigated the associations between SNPs of inflammatory molecules, consisting of IL-1β, TNF-α, IL-6, IL-10, IL-18, interferon-γ (IFN-γ) and C-reactive protein (CRP), and PSD at 2 weeks after stroke (early-onset PSD). We also analyzed whether splenic attenuation (SA) on computed tomography (CT) [reflecting splenic density, because attenuation can be estimated by the physical density of an organ (22)], SV on CT and peripheral immuno-inflammatory parameters have an association with early-onset PSD.

## Materials and methods

### Recruitment

We recruited 103 stroke patients admitted to the Department of Neurology, Xiangya Hospital of Centre South University from July 2019 to August 2021. All patients met the following inclusion criteria (1): age between 18 and 75 years (2); within 2 weeks after stroke; (3) diagnosed ischemic stroke or intracerebral hemorrhage by brain CT or magnetic resonance imaging; (4) conducted an unenhanced chest CT scan on which the intact spleen can be clearly shown; (5) ability to complete all necessary investigations and questionnaires; and (6) capacity to provide informed consent. The exclusion criteria were as follows: patients with any self-report psychiatric illness (previous treatment or clinical diagnose); any comorbid neuropsychiatric conditions, particularly dementia, Parkinson’s disease, brain tumor, epilepsy, psychoses, and alcohol or substance dependence; severe aphasia or dysarthria, visual or auditory impairment; hematopoietic diseases; splenic lesions; malignant lesions; serious or longstanding infection; liver diseases or portal hypertension; autoimmune diseases; metabolism diseases. The study was approved by “Medical Ethics Committee of the Xiangya Hospital of Centre South University”.

### Participant characteristics

The characteristics potentially in connection with PSD or functional status were collected as covariates: age, gender, body mass index (BMI, defined as the body mass divided by the square of the body height), years of education, pulmonary infection, medicines (antibiotic, statin and antiplatelet agents), vascular risk factors (hypertension, diabetes mellitus, heart disease, hyperlipidemia, smoking, drinking and previous stroke), stroke type (ischemic, hemorrhagic and subtypes based on TOAST classification), stroke hemisphere (left, right or bilateral), stroke location (anterior, posterior or both), stroke severity assessed by the National Institutes of Health and Stroke Scale (NIHSS), cognitive function evaluated by the Mini-Mental State Examination (MMSE), the times from stroke onset to chest CT scan and to the blood sample collection. Complete blood count parameters including leukocyte, neutrophil, monocyte, lymphocyte and platelet counts were obtained from the blood routine results at admission and platelet-to-lymphocyte ratio, neutrophil-to-lymphocyte ratio and monocyte-to-lymphocyte ratio were calculated. All stroke patients were assessed for depressive symptoms at 2 weeks after onset by Diagnostic and Statistical Manual of Mental Disorders, 5th edition (DSM-V, American Psychiatric Association, 2013) and then classified into PSD and non-PSD groups. All investigators had undergone professional pre-job training according to the depression diagnostic guideline.

### Spleen measurements

**Figure 1:**
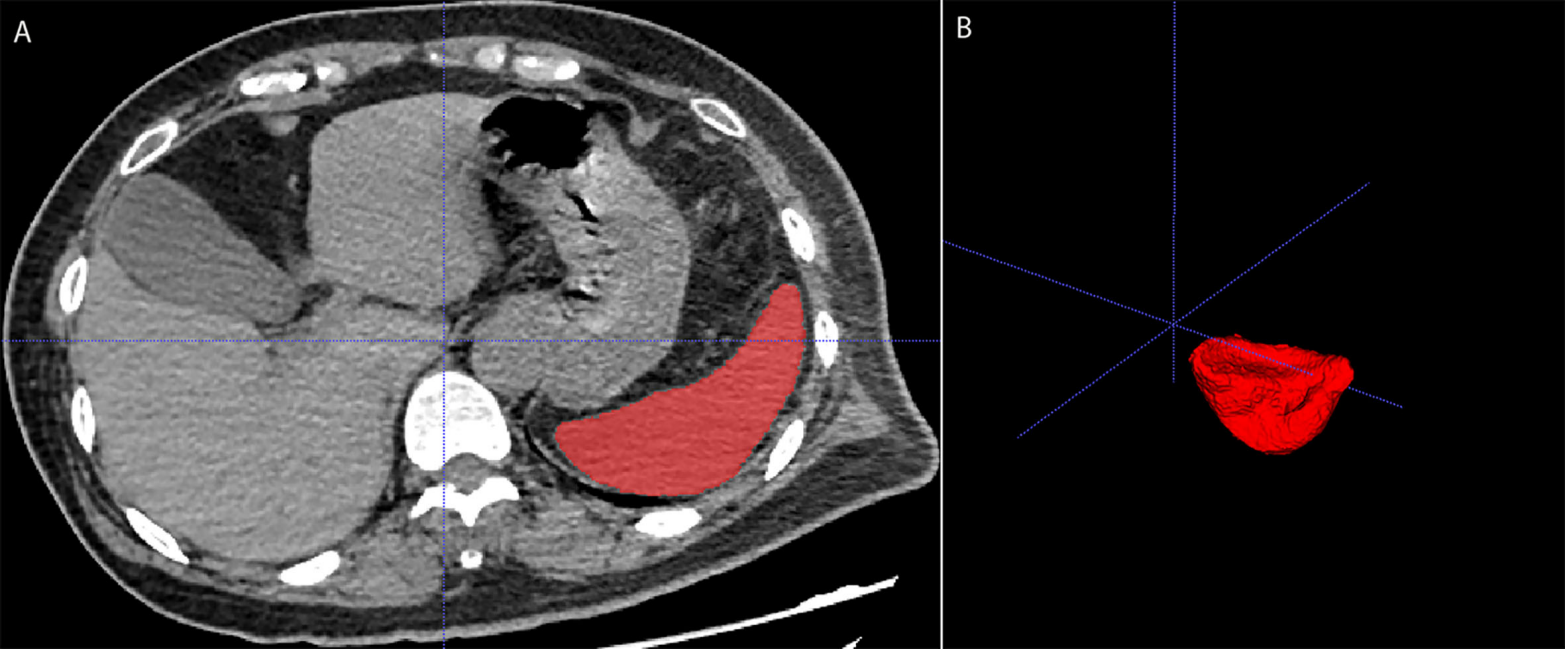
Measurement of splenic volume on unenhanced chest computed tomography using ITK-SNAP software. **(A)** showed the spleen segmentation, while **(B)** presented 3D model of the spleen.

**Figure 2:**
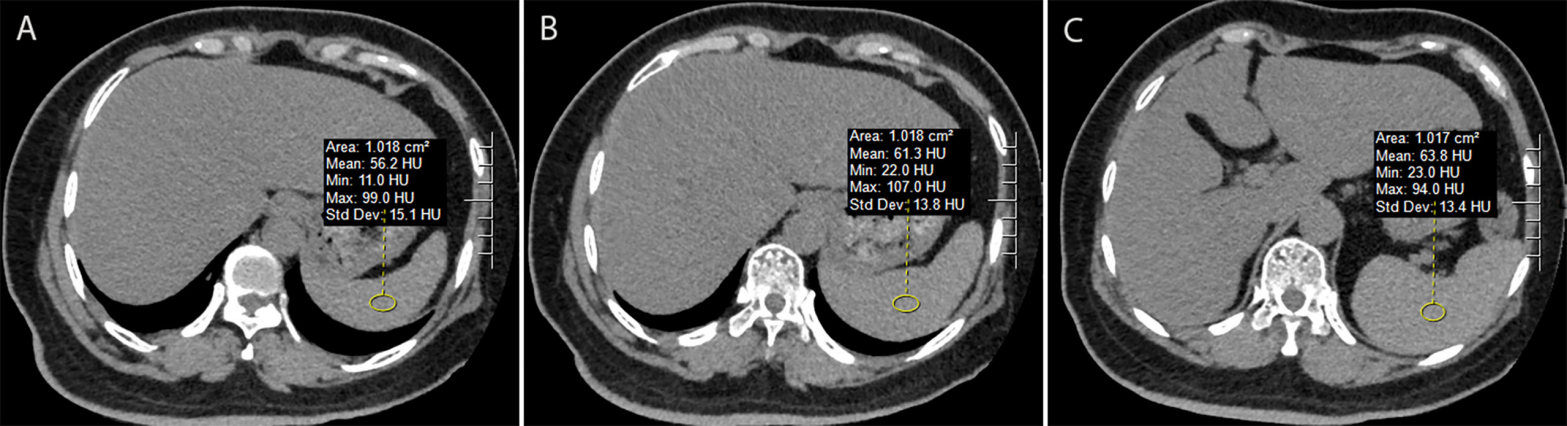
Measurement of splenic attenuation (SA) on unenhanced chest computed tomography using imaging Picture Archiving and Communication System. Three circular region-of-interests (about 1.0 cm^2^ in size) were placed in the upper **(A)**, middle **(B)**, and lower third **(C)** of the spleen respectively. The average of these three measurements was used for the SA value.

CT is viewed as the most reliable noninvasive imaging technique for the *in vivo* assessment of SV (23). All subjects completed a high resolution (512×512 image matrix, 1.25-mm slice thickness) non-enhance chest CT scan (Aqulion one). The spleen was segmented on CT using a three-dimensional volumetric analysis software (ITK-SNAP) to calculate SV (**Figure 1**) (24). SA was obtained by averaging the three Hounsfield units for about 1.0 cm^2^ circular region-of-interest placed in the upper, middle, and lower thirds of the spleen, on imaging Picture Archiving and Communication System (version 4.1.3.0) (**Figure 2**). The method of measuring SA has been reported (25, 26). All spleen measurements were performed by a radiologist with five years’ experience.

### SNPs selection and gene sequencing

In accordance with information in the National Center for Biotechnology Information SNP database (http://www.ncbi.nlm.nih.gov/SNP) and the Chinese dataset of an online genetic database (http://www.ensembl.org), we screened up to 22 SNPs (with a minor allele frequency of > 5%) of IL-1β, TNF-α, IL-6, IL-10, IL-18, IFN-γ and CRP candidate genes previously reported to be associated with depression, stroke or PSD. Samples for testing polymorphisms were extracted from admission venous blood using standard procedures. The SNPs were genotyped by SNPscan™ multiplex SNP typing kit (Cat#: G0104K, Genesky Biotechnologies Inc. Shanghai, China), provided by Shanghai Tianhao Biotechnology Co., Ltd., China. The SNP typing technique has been reported in the study of Cheng et al. (27). 5% of duplicate DNA samples were analyzed for quality control, with consistence in more than 99% of the samples.

### Statistical analysis

Statistical analysis was done using SPSS 26.0 statistical package. The normality of continuous variables was examined by Shapiro-Wilk test. Results were presented as percentages for enumeration data compared using Chi-squared test or Fisher’s exact test, as means ± standard deviation for continuous variables with normal distributions compared using Student’s t-test, and as median (interquartile range) for continuous variables with skewed distributions compared using Mann–Whitney U-test. The genotype distributions of SNPs were assessed by Hardy–Weinberg equilibrium (HWE) using the Chi-square test. SNPs allele frequencies between PSD and non-PSD groups were also evaluated by Chi-squared test. Associations of genotypes with PSD (by comparing genotype distributions with non-PSD) were analyzed with binary logistic regression model. SA and SV were analyzed by Student’s t-test or Mann–Whitney U-test between PSD and non-PSD groups. Complete blood count parameters were assessed by Mann–Whitney U-test between PSD and non-PSD groups. A *p* value of less than 0.05 was considered as statistically significant.

## Results

### Characteristics of PSD and non-PSD patients

Demographic and clinical characteristics of 103 participants were summarized (**Table 1**). Participants had a median (interquartile range) age of 57 (53–67) years and 69.9% were male, 85 patients with ischemic stroke and 18 patients with intracerebral hemorrhage. 49 out of the 103 stroke patients were diagnosed as early-onset PSD (47.6%).

**Table 1:** Demographic and clinical characteristics of PSD and non-PSD patients.

	PSD, n = 49	Non-PSD, n = 54	*P* value
Age (years)	60 (54-69)	57 (52-66)	0.052
Gender: female	20 (40.80)	11 (20.37)	0.024
BMI (kg/m^2^)	23.66 (22.06-25.29)	23.63 (22.04-24.93)	0.947
Years of education	9 (6-12)	12 (9-16)	0.044
Pulmonary infection	16 (32.65)	14 (25.93)	0.453
Antibiotic	9 (18.37)	10 (18.52)	0.984
Statin	42 (85.71)	46 (85.19)	0.939
Antiplatelet	39 (79.59)	46 (85.19)	0.455
Vascular risk factors			
Hypertension	40 (81.65)	46 (85.19)	0.628
Diabetes mellitus	16 (32.65)	16 (29.63)	0.741
Heart disease	5 (10.20)	4 (7.41)	0.733
Hyperlipidemia	24 (48.98)	25 (46.30)	0.785
Smoking	21 (42.86)	30 (55.56)	0.198
Drinking	23 (46.94)	29 (53.70)	0.490
Previous stroke	6 (12.25)	5 (9.26)	0.624
Stroke type: ischemic stroke	39 (79.59)	46 (85.19)	0.455
Large-artery atherosclerosis	23 (58.98)	36 (78.26)	0.145
Cardioembolism	3 (7.69)	1 (2.17)	
Small-vessel occlusion	2 (5.13)	3 (6.52)	
Other determined etiology	1 (2.56)	2 (4.35)	
Undetermined etiology	10 (25.64)	4 (8.70)	
Stroke hemisphere			0.141
Left	13 (26.53)	23 (42.59)	
Right	34 (69.39)	27 (50.00)	
Bilateral	2 (4.08)	4 (7.41)	
Stroke location			0.193
Anterior	35 (71.43)	29 (53.70)	
Posterior	11 (22.45)	19 (35.19)	
Both	3 (6.12)	6 (11.11)	
NIHSS score	5 (1.5-8.5)	2 (1-4.5)	0.046
MMSE score	24 (22-27)	27 (23-29)	0.027
Onset to chest CT scan (days)	4 (1-9)	5 (2-7)	0.705
Onset to blood sample collection (days)	2 (1-5)	2 (0.75-5)	0.930
Leukocyte count (10^9^/L)	7.10 (6.00-8.30)	7.65 (6.18-9.00)	0.176
Neutrophil count (10^9^/L)	5.00 (3.80-6.10)	4.90 (3.68-6.83)	0.726
Monocyte count (10^9^/L)	0.50 (0.35-0.60)	0.50 (0.48-0.70)	0.007
Lymphocyte count (10^9^/L)	1.30 (1.10-1.60)	1.50 (1.20-2.00)	0.052
Platelet count (10^9^/L)	197.00 (156.50-239.00)	217.00 (176.75-253.25)	0.193
Platelet/lymphocyte	142.67 (113.90-203.48)	140.53 (102.27-192.71)	0.422
Neutrophil/lymphocyte	3.54 (2.45-5.43)	3.33 (2.18-5.19)	0.472
Monocyte/lymphocyte	0.33 (0.22-0.48)	0.33 (0.26-0.48)	0.492
SA (HU)	47.68 (43.99-54.53)	49.05 (47.15-55.79)	0.060
SV (cm^3^)	150.40 (117.45-192.10)	130.85 (91.59-173.35)	0.153

Values are n (%) or median (interquartile range). PSD, post-stroke depression; BMI, body mass index; NIHSS, National Institutes of Health and Stroke Scale; MMSE, Mini-Mental State Examination; CT, computed tomography; SA, splenic attenuation; SV, splenic volume; HU, Hounsfield unit.

We observed that compared with the non-PSD group, PSD group showed a higher proportion of female (40.80% vs. 20.37%, *p* = 0.024), lower years of education (9 [6-12] vs. 12 [9-16]; *p* = 0.044), higher stroke severity (NIHSS score: 5 [1.5-8.5] vs. 2 [1-4.5]; *p* = 0.046), worse cognitive function (MMSE score: 24 [22-27] vs. 27 [23-29]; *p* = 0.027) and lower monocyte count (0.50 [0.35-0.60] vs. 0.50 [0.48-0.70]; *p* = 0.007). BMI, pulmonary infection, medicines (antibiotic, statin and antiplatelet agents), vascular risk factors (hypertension, diabetes mellitus, heart disease, hyperlipidemia, smoking, drinking and previous stroke), stroke type including TOAST classification, stroke hemisphere, stroke location, the times from stroke onset to chest CT scan and to the blood sample collection, complete blood count parameters (leukocyte, neutrophil, lymphocyte and platelet counts, platelet-to-lymphocyte ratio, neutrophil-to-lymphocyte ratio and monocyte-to-lymphocyte ratio), and SV did not show any significantly association with PSD status.

### Polymorphisms of IL-1β, TNF-α, IL-6, IL-10, IL-18, IFN-γ and CRP

Genotype distributions and allele frequencies of the seven selected inflammatory molecule polymorphisms in PSD and non-PSD groups were documented (**Tables 1**, **2** in the **Supplementary Material**). The call rates of all SNPs were 100%. All genotypes were in HWE (*p* > 0.05). For CRP gene, the C/T genotypes of rs2794520 and rs1205 were significantly more in PSD group than non-PSD group (both odds ratio [OR] = 3.229; 95%CI: 1.090-9.570; *p* = 0.034). The remaining 20 SNPs genotypes showed no statistical difference between the two groups (*p* > 0.05). Of all alleles frequencies for the 22 SNPs described here, only CRP rs3091244 allele frequency had statistical significance (χ^2^ = 104.380; *p* < 0.001), showing that G allele was significantly higher (80.61% vs. 13.89%) while A allele was significantly less (6.12% vs. 71.30%) in PSD patients compared to non-PSD patients. These findings indicated that CRP rs2794520 C/T genotype, rs1205 C/T genotype and rs3091244 G allele was related to an increase in the risk of PSD while rs3091244 A allele was associated with a reduction in the risk of early-onset PSD.

### Independent risk factors of early-onset PSD

We further analyzed the independent risk factors of PSD (**Table 2**). The C/T genotypes of CRP rs2794520 and rs1205 between PSD and non-PSD groups still had significant differences after gender, years of education, NIHSS score, MMSE score and monocyte count adjustments (both adjusted OR = 3.364; 95%CI: 1.039-10.898; *p* = 0.043). There were also significant differences in monocyte count between PSD and non-PSD groups after adjusting for other variables (adjust OR = 0.057; 95%CI: 0.005-0.686; *p* = 0.024). CRP rs2794520 C/T genotype, rs1205 C/T genotype and lower monocyte count were independent risk factors of early-onset PSD.

**Table 2:** Independent risk factors of PSD.

Variables	Unadjusted		Adjusted	
	OR (95%CI)	*P* value	OR (95%CI)	*P* value
Gender				
Male	Ref	–	Ref	–
Female	2.696 (1.125-6.458)	0.026	1.980 (0.645-6.081)	0.233
Years of education	0.905 (0.814-1.007)	0.067	0.980 (0.845-1.137)	0.790
NIHSS score	1.117 (1.006-1.241)	0.039	1.122 (0.998-1.262)	0.055
MMSE score	0.944 (0.866-1.030)	0.195	0.998 (0.891-1.118)	0.972
Monocyte count (10^9^/L)	0.039 (0.004-0.417)	0.007	0.057 (0.005-0.686)	0.024
CRP rs2794520				
C/C	Ref	–	Ref	–
C/T	3.229 (1.090-9.570)	0.034	3.364 (1.039-10.898)	0.043
T/T	2.000 (0.594-6.730)	0.263	2.161 (0.579-8.061)	0.251
CRP rs1205				
C/C	Ref	–	Ref	–
C/T	3.229 (1.090-9.570)	0.034	3.364 (1.039-10.898)	0.043
T/T	2.000 (0.594-6.730)	0.263	2.161 (0.579-8.061)	0.251

PSD, post-stroke depression; OR, odds ratio; CI, confidence interval; NIHSS, National Institutes of Health and Stroke Scale; MMSE, Mini-Mental State Examination; CRP, C-reactive protein.

### SA and SV based on CRP genotypes

The SA and SV based on the genotype of CRP rs2794520 and rs1205 were compared between PSD and non-PSD group (**Table 3**). We found that SA was significantly lower in PSD group than that in non-PSD group in the presence of CRP rs2794520 C/T genotype and rs1205 C/T genotype (both t = 2.122; *p* = 0.039). But there was no significant difference in SV between PSD and non-PSD groups (*p* > 0.05)

**Table 3:** SA and SV by CRP SNP genotypes and PSD status.

SNP genotype	PSD	Non-PSD	z/t	*P* value
Rs2794520 C/C, SA SV C/T, SA SV T/T, SA SV	51.82 (44.78-58.20) 147.65 (129.35-192.53) 48.19 ± 5.69 136.60 (109.40-180.40) 50.04 ± 5.11 188.21 ± 25.64	48.63 (47.10-58.60) 124.30 (93.46-187.60) 51.64 ± 6.34 130.85 (87.86-199.70) 50.07 ± 5.10 131.69 ± 11.55	z = -0.467 z = -1.090 t = 2.122 z = -0.025 t = 0.015 t = -2.010	0.640 0.276 0.039 0.980 0.988 0.062
Rs1205 C/C, SA SV C/T, SA SV T/T, SA SV	51.82 (44.78-58.20) 147.65 (129.35-192.53) 48.19 ± 5.69 136.60 (109.40-180.40) 50.04 ± 5.11 188.21 ± 25.64	48.63 (47.10-58.60) 124.30 (93.46-187.60) 51.64 ± 6.34 130.85 (87.86-199.70) 50.07 ± 5.10 131.69 ± 11.55	z = -0.467 z = -1.090 t = 2.122 z = -0.025 t = 0.015 t = -2.010	0.640 0.276 0.039 0.980 0.988 0.062

Values are means ± standard deviation or median (interquartile range). SA, splenic attenuation; SV, splenic volume; CRP, C-reactive protein; SNP, single nucleotide polymorphism; PSD, post-stroke depression.

## Discussion

This is the first study focusing on the association between immuno-inflammatory response and early-onset PSD from the microscopic level in SNPs of inflammatory molecules and the macroscopic level in peripheral inflammation-related parameters, SA and SV. The principal findings here were (1) that the C/T genotypes of CRP rs2794520 and rs1205, and CRP rs3091244 G allele were associated with an increased risk of early-onset PSD, while CRP rs3091244 A allele was associated with a reduced risk of early-onset PSD; (2) and that SA was correlated with the risk of early-onset PSD in the presence of CRP rs2794520 C/T genotype and rs1205 C/T genotype; (3) and that peripheral lower monocyte count was related to an increased risk of early-onset PSD. Our findings indicate from different points of view that activated immune system characterized by a widespread inflammatory response, where stroke act as a precipitating factor, plays an important role in the occurrence of early-onset PSD.

To our knowledge, there to date were only two studies emphasizing the effect of inflammatory molecule polymorphisms on PSD liability. Kim et al. first reported the association of cytokine polymorphisms with PSD, showing that patients with polymorphisms of anti-inflammatory cytokines IL-4 and IL-10 rather than pro-inflammatory cytokines TNF-α, IL-1β, IL-6 and IL-8 had an increased propensity to develop depression in the acute phase of stroke (28). Another stroke cohort by Kim et al. showed that TNF-α -850T allele and IL-1β –511T allele were not independently associated with PSD status, but the interaction with cytokine levels played an important role in the risk of PSD at 2 weeks (29). We did not observe, however, polymorphisms of pro-inflammatory cytokines (IL-1β, TNF-α, IL-6, IL-18 and IFN-γ) and anti-inflammatory cytokines (IL-10) endowed pronounced impacts on the risk of early-onset PSD. This could indicate that the association of polymorphisms in pro- and anti-inflammatory cytokines with early-onset PSD is either not causal or is influenced by pleiotropy. The racial heterogeneity of cytokine SNPs (30), methodological differences and choice of subjects could give rise to the discrepancy in early-onset PSD genetic risks among studies.

Interestingly, we found that the C/T genotypes of CRP rs2794520 and rs1205 were associated with an increased occurrence of early-onset PSD even after gender, years of education, NIHSS score, MMSE score and monocyte count adjustments, which were independent risk factors for early-onset PSD. We observed, moreover, that CRP rs3091244 G allele was a predisposing factor for early-onset PSD, while A allele was a protective factor against early-onset PSD. CRP is traditionally classed as a biomarker of both peripheral and central inflammation (31), but there is converging evidence that CRP plays a significant role in regulating and amplifying inflammatory process (32, 33). Serum CRP level is influenced by specific SNPs including rs2794520, rs1205 and rs3091244 (34–36). Recently, Otsuka et al. pointed out that CRP SNPs might engender elevated levels of multiple inflammatory molecules comprising cytokines and CRP, thereby resulting in neuroinflammation (37), a critical process in the pathogenesis of PSD (38). Besides, a latest meta-analysis underscored that a higher level of CRP in the acute phase of stroke predicted an augmented risk of PSD (39). On the basis of these evidences, the possible explanation for the associations in current study is that CRP polymorphisms may be involved in the occurrence of depression following stroke by causing changes in inflammatory molecule levels, which affect neurotransmitters, hormones or other mechanisms in relation to depression (9).

The framework of inflammation for depressive symptoms after stroke has, recently, garnered amount of attention. As one of the primary peripheral immune organs, spleen is a key participant in the immuno-inflammatory response in response to stroke. The activation of spleen after stroke is predominantly associated with the following events that are paramount for the initiation of effective brain-spleen crosstalk: the interactions of chemokines egress from damaged brain cells with chemokine receptors upregulated by splenocytes, autonomic nervous system activation, the secretion of antigens from compromised brain (40, 41). Concomitant with spleen activation after stroke, the changes of it are mainly manifested in morphology, the numbers of immunocytes and cytokines production, which may be involved in the progression of early-onset PSD by potentiating neuroinflammation. The data, however, showed here that there was no association between SV and PSD status, but lower SA was significantly associated with PSD status in the presence of CRP rs2794520 C/T genotype and rs1205 C/T genotype. The mechanisms underlying the associations may be that owing to the contribution of CRP polymorphism and CRP levels to immuno-inflammatory process (32, 37), patients with the C/T genotypes of rs2794520 and rs1205 may be in a more active inflammatory state so that immune cells and cytokines produced abundantly in the spleen, in response to brain vascular damage, were released into the bloodstream and migrated to the site of brain insult (17, 20), aggravating secondary brain inflammatory response which influences serotonin metabolism and causes noradrenergic system and HPA axis imbalance, may culminate in PSD (6, 42). There in addition were evidence from animal studies for the involvement of pro- and anti-inflammatory cytokines produced by splenic cells in the formation of depression like behavior (43, 44). So lower SA in early-onset PSD patients with genetic susceptibility to CRP might be a consequence of more intense splenic activities. Our results suggested that SA may be a potential biomarker of early-onset PSD, further supported the immuno-inflammatory mechanism in etiology of PSD from an image-based perspective.

Among circulating immuno-inflammatory parameters in present study, only monocyte count was independently associated with the risk of early-onset PSD. The plethora of strong evidence have corroborated that monocyte can rapidly trafficking from spleen to the site of brain insult and regulate neuroimmune responses (45–47), facilitating the development of depressive-like behavior (48). Thus, the lower monocyte count of early-onset PSD patients may be the result of excessive migration of monocytes to cerebral injury site. Hu et al. proposed that higher neutrophil-to-lymphocyte ratio and platelet-to-lymphocyte ratio were correlated with PSD at 6 months (49), and Ding et al. found that monocyte-to-lymphocyte ratio was in association with depression 3 months after stroke (50). But these associations were not shown in current study. The inconsistency may be attributed to the times from onset to depression assessment and to blood samples collection.

Albeit with these promising findings in our study, there are some limitations need to be considered. Firstly, not all the SNP locis of inflammatory molecules were tested and large-scale genome-wide association studies may identify more genetic risk SNP locis associated with early-onset PSD. Secondly, study on the associations of inflammatory molecular polymorphisms with PSD susceptibility was conducted in the Chinese population and investigations are warranted in different ethnicities. Thirdly, this was a cross-sectional study underscoring inflammatory risks of early-onset PSD and further longitudinal study on the association between immuno-inflammatory response and the risk of PSD may contribute to clarify whether immuno-inflammatory activities are involved in the occurrence of PSD in a time-dependent manner.

In conclusion, the findings of the current study suggest the important role of peripheral immuno-inflammatory response in the risk of early-onset PSD. Characteristics of the splenic activities after acute stroke and their contribution to the occurrence of depression at 2 weeks after stroke may provide new insights for assessment and recognition of early-onset PSD. Future developments should struggle to elucidate the pathophysiology of PSD on immuno-inflammatory mechanisms, furthering our understanding in the common neuropsychiatric sequelae of stroke, especially in the absence of specific and sensitive biomarkers for the diagnosis of PSD (51).

## Data availability statement

The data presented in the study are deposited in the European Nucleotide Archive (ENA) repository, accession number PRJEB56038.

## Ethics statement

The studies involving human participants were reviewed and approved by “Medical Ethics Committee of the Xiangya Hospital of Centre South University”. The patients/participants provided their written informed consent to participate in this study

## Author contributions

HC, FL, FT designed the study and drafted the manuscript. HC, FL, DS, JZ, SL, QL, FT collected and analyzed the data, and reviewed the manuscript. All authors contributed to the article and approved the submitted version.

## Funding

This work was supported by National key Research and Development Program of China (No. 2017YFC1310003).

## Acknowledgments

All authors thank every volunteer who agreed to participate in the trial.

## Conflict of interest

The authors declare that the research was conducted in the absence of any commercial or financial relationships that could be construed as a potential conflict of interest.

## Publisher’s note

All claims expressed in this article are solely those of the authors and do not necessarily represent those of their affiliated organizations, or those of the publisher, the editors and the reviewers. Any product that may be evaluated in this article, or claim that may be made by its manufacturer, is not guaranteed or endorsed by the publisher.
