# Supplementary material for: The potential risk factors of early-onset post-stroke depression from immuno-inflammatory perspective
Source: Front Immunol. 2022 Sep 26;13:1000631. doi: 10.3389/fimmu.2022.1000631 (PMC9549963; doi:10.3389/fimmu.2022.1000631)
Supplement: Supplementary file 1 [file Table_1.docx]

Supplementary Material

# Supplementary Tables

**Table 1** **| Genotype and allele frequencies in PSD and non-PSD patients.**

| **Gene** | **SNP** | **Genotype or**  **allele** | **PSD, n (%)** | **Non-PSD, n (%),** | **OR (95%CI) or χ^2^** | ***P* value** |
| --- | --- | --- | --- | --- | --- | --- |
| IL-1β | rs1143623 | C/C | 16 (32.65) | 19 (35.18) | Ref | - |
|  |  | C/G | 23 (46.94) | 25 (46.30) | 1.092 (0.456-2.617) | 0.843 |
|  |  | G/G | 10 (20.41) | 10 (18.52) | 1.187 (0.395-3.568) | 0.760 |
|  |  | C | 55 (56.12) | 63 (58.33) | 0.103 | 0.749 |
|  |  | G | 43 (43.88) | 45 (41.67) |  |  |
| IL-1β | rs1143627 | G/G | 12 (24.49) | 12 (22.22) | Ref | - |
|  |  | G/A | 26 (53.06) | 26 (48.15) | 1.000 (0.380-2.631) | 1.000 |
|  |  | A/A | 11 (22.45) | 16 (29.63) | 0.508 (0.227-2.084) | 0.508 |
|  |  | G | 50 (51.02) | 50 (46.30) | 0.459 | 0.498 |
|  |  | A | 48 (48.98) | 58 (53.70) |  |  |
| IL-1β | rs1143634 | G/G | 49 (1.00) | 53 (98.15) | Ref | - |
|  |  | G/A | 0 (0.00) | 1 (1.85) | - | - |
|  |  | G | 98 (100.00) | 107 (99.07) | - | - |
|  |  | A | 0 (0) | 1 (0.93) |  |  |
| IL-1β | rs1143643 | C/C | 13 (26.53) | 10 (18.52) | Ref | - |
|  |  | C/T | 25 (51.02) | 28 (51.85) | 0.687 (0.256-1.839) | 0.455 |
|  |  | T/T | 11 (22.45) | 16 (29.63) | 0.529 (0.171-1.631) | 0.268 |
|  |  | C | 51 (52.04) | 48 (44.44) | 1.188 | 0.276 |
|  |  | T | 47 (47.96) | 60 (55.56) |  |  |
| TNF-α | rs1799724 | C/C | 37 (75.51) | 46 (85.19) | Ref | - |
|  |  | C/T | 10 (20.41) | 8 (14.81) | 1.554 (0.557-4.333) | 0.399 |
|  |  | T/T | 2 (4.08) | 0 (0.00) | - | - |
|  |  | C | 84 (85.71) | 100 (92.59) | 2.548 | 0.110 |
|  |  | T | 14(14.29) | 8(7.41) |  |  |
| TNF-α | rs1799964 | T/T | 34 (69.39) | 37 (68.52) | Ref | - |
|  |  | T/C | 14 (28.57) | 13 (24.07) | 1.172 (0.483-2.845) | 0.726 |
|  |  | C/C | 1 (2.04) | 4 (7.41) | 0.272 (0.029-2.556) | 0.255 |
|  |  | T | 82(83.67) | 87(80.56) | 0.339 | 0.560 |
|  |  | C | 16(16.33) | 21(19.44) |  |  |
| TNF-α | rs1800629 | G/G | 46 (93.88) | 46 (85.19) | Ref | - |
|  |  | G/A | 3 (6.12) | 7 (12.96) | 0.429 (0.104-1.761) | 0.240 |
|  |  | A/A | 0 (0.00) | 1 (1.85) | - | - |
|  |  | G | 95(96.94) | 99(91.67) | 2.603 | 0.107 |
|  |  | A | 3(3.06) | 9(8.33) |  |  |
| TNF-α | rs361525 | G/G | 45 (91.84) | 52 (96.30) | Ref |  |
|  |  | G/A | 4 (8.16) | 2 (3.70) | 2.311 (0.404-13.215) | 0.346 |
|  |  | G | 94 (95.92) | 106 (98.15) | - | 0.427 |
|  |  | A | 4 (4.08) | 2 (1.85) |  |  |
| IL-6 | rs1800795 | C/G | 0 (0.00) | 1 (1.85) | Ref | - |
|  |  | G/G | 49 (1.00) | 53 (98.15) | - | - |
|  |  | C | 0 (0.00) | 1 (0.93) | - | - |
|  |  | G | 98 (100.00) | 107 (99.07) |  |  |
| IL-6 | rs2069824 | T/T | 49 (1.00) | 54 1.00) | - | - |
| IL-10 | rs1800872 | T/T | 20 (40.82) | 28 (51.85) | Ref | - |
|  |  | T/G | 24 (48.98) | 20 (37.04) | 1.680 (0.736-3.835) | 0.218 |
|  |  | G/G | 5 (10.20) | 6 (11.11) | 1.167 (0.312-4.360) | 0.819 |
|  |  | T | 64 (65.31) | 76 (70.37) | 0.605 | 0.437 |
|  |  | G | 34 (34.69) | 32 (29.63) |  |  |
| IL-10 | rs1800896 | T/T | 43 (87.76) | 49 (90.74) | Ref | - |
|  |  | T/C | 6 (12.24) | 5 (9.26) | 1.367 (0.390-4.799) | 0.625 |
|  |  | T | 92 (93.88) | 103 (95.37) | 0.227 | 0.634 |
|  |  | C | 6 (6.12) | 5 (4.63) |  |  |
| IL-18 | rs1946518 | T/T | 10 (20.41) | 13 (24.07) | Ref | - |
|  |  | T/G | 22 (44.90) | 25 (46.30) | 1.144 (0.419-3.122) | 0.793 |
|  |  | G/G | 17 (34.69) | 16 (29.63) | 1.381 (0.474-4.028) | 0.554 |
|  |  | T | 42 (42.86) | 51 (47.22) | 0.395 | 0.530 |
|  |  | G | 56 (57.14) | 57 (52.78) |  |  |
| IL-18 | rs543810 | C/C | 7 (14.29) | 6 (11.11) | Ref | - |
|  |  | C/T | 16 (32.65) | 23 (42.59) | 0.596 (0.169-2.109) | 0.422 |
|  |  | T/T | 26 (53.06) | 25 (46.30) | 0.891 (0.263-3.022) | 0.854 |
|  |  | C | 30 (30.61) | 35 (32.41) | 0.077 | 0.782 |
|  |  | T | 68 (69.39) | 73 (67.59) |  |  |
| IFN-γ | rs1861494 | C/C | 7 (14.29) | 7 (12.97) | Ref | - |
|  |  | C/T | 18 (36.73) | 24 (44.44) | 0.750 (0.223-2.522) | 0.642 |
|  |  | T/T | 24 (48.98) | 23 (42.59) | 1.043 (0.316-3.442) | 0.944 |
|  |  | C | 32 (32.65) | 38 (35.18) | 0.147 | 0.702 |
|  |  | T | 66 (67.35) | 70 (64.82) |  |  |
| CRP | rs2794520 | C/C | 6 (12.24) | 15 (27.78) | Ref | - |
|  |  | C/T | 31 (63.27) | 24 (44.44) | 3.229 (1.090-9.570) | 0.034 |
|  |  | T/T | 12 (24.49) | 15 (27.78) | 2.000 (0.594-6.730) | 0.263 |
|  |  | C | 43 (43.88) | 54 (50.00) | 0.773 | 0.379 |
|  |  | T | 55 (56.12) | 54 (50.00) |  |  |
| CRP | rs1417938 | T/T | 47 (95.92) | 50 (92.59) | Ref | - |
|  |  | T/A | 2 (4.08) | 4 (7.41) | 0.532 (0.093-3.041) | 0.478 |
|  |  | T | 96 (97.96) | 104 (96.30) | - | 0.685 |
|  |  | A | 2 (2.04) | 4 (3.70) |  |  |
| CRP | rs1205 | C/C | 6 (12.24) | 15 (27.78) | Ref | - |
|  |  | C/T | 31 (63.27) | 24 (44.44) | 3.229 (1.090-9.570) | 0.034 |
|  |  | T/T | 12 (24.49) | 15 (27.78) | 2.000 (0.594-6.730) | 0.260 |
|  |  | C | 43 (43.88) | 54 (50.00) | 0.773 | 0.379 |
|  |  | T | 55 (56.12) | 54 (50.00) |  |  |
| CRP | rs11265260 | A/A | 33 (67.35) | 29 (53.71) | Ref | - |
|  |  | A/G | 14 (28.57) | 24 (44.44) | 0.513 (0.224-1.172) | 0.113 |
|  |  | G/G | 2 (4.08) | 1 (1.85) | 1.758 (0.151-20.403) | 0.652 |
|  |  | A | 80 (81.63) | 82 (75.93) | 0.996 | 0.318 |
|  |  | G | 18 (18.37) | 26 (24.07) |  |  |
| CRP | rs1130864 | G/G | 47 (95.92) | 50 (92.59) | Ref | - |
|  |  | G/A | 2 (4.08) | 4 (7.41) | 0.532 (0.093-3.041) | 0.478 |
|  |  | G | 96 (97.96) | 104 (96.30) | - | 0.685 |
|  |  | A | 2 (2.04) | 4 (3.70) |  |  |
| CRP | rs3093059 | A/A | 32 (65.31) | 29 (53.71) | Ref | - |
|  |  | A/G | 15 (30.61) | 24 (44.44) | 0.566 (0.250-1.283) | 0.173 |
|  |  | G/G | 2 (4.08) | 1(1.85) | 1.812 (0.156-21.056) | 0.635 |
|  |  | A | 79 (80.61) | 82 (75.93) | 0.661 | 0.416 |
|  |  | G | 19 (19.39) | 26 (24.07) |  |  |
| CRP | rs3091244 | G/G | 30 (61.23) | 28 (51.85) | Ref | - |
|  |  | G/A | 2 (4.08) | 1 (1.85) | 1.867 (0.160-21.742) | 0.618 |
|  |  | G/T | 15 (30.61) | 21 (38.89) | 0.667 (0.288-1.543) | 0.344 |
|  |  | T/A | 0 (0.00) | 3 (5.56) | - | - |
|  |  | T/T | 2 (4.08) | 1 (1.85) | 1.867 (0.160-21.742) | 0.618 |
|  |  | G | 79 (80.61) | 15 (13.89) | 104.380 | ＜0.001 |
|  |  | A | 6 (6.12) | 77 (71.30) |  |  |
|  |  | T | 13 (13.27) | 16 (14.81) |  |  |
